# Prevalence of frailty in severe mental illness: findings from the UK Biobank

**DOI:** 10.1192/bjo.2023.580

**Published:** 2023-10-12

**Authors:** Nicola Warren, Stuart Leske, Urska Arnautovska, Korinne Northwood, Steve Kisely, Dan Siskind

**Affiliations:** Faculty of Medicine, The University of Queensland, Brisbane, Australia; and Metro South Addiction and Mental Health Service, Metro South Health, Brisbane, Australia; Faculty of Medicine, The University of Queensland, Brisbane, Australia

**Keywords:** Frailty, schizophrenia, bipolar, depression, severe mental illness

## Abstract

**Background:**

Severe mental illness (SMI) is associated with significant morbidity. Frailty combines biological ageing, comorbidity and psychosocial factors and can predict adverse health outcomes. Emerging evidence indicates that frailty is higher in individuals with SMI than in the general population, although studies have been limited by sample size.

**Aims:**

To describe the prevalence of frailty in people with SMI in a large cohort using three different frailty measures and examine the impact of demographic and sociodemographic variables.

**Method:**

The UK Biobank survey data, which included individuals aged 37–73 years from England, Scotland and Wales from 2006 to 2010, with linked in-patient hospital episodes, were utilised. The prevalence of frailty in individuals with and without SMI was assessed through three frailty measures: frailty index, physical frailty phenotype (PFP) and Hospital Frailty Risk Score (HFRS). Stratified analysis and dichotomous logistic regression were conducted.

**Results:**

A frailty index could be calculated for 99.5% of the 502 412 UK Biobank participants and demonstrated greater prevalence of frailty in women and an increase with age. The prevalence of frailty for those with SMI was 3.19% (95% CI 3.0–3.4), 4.2% (95% CI 3.8–4.7) and 18% (95% CI 15–23) using the frailty index, PFP and HFRS respectively. The prevalence ratio was between 3 and 18 times higher than in those without SMI.

**Conclusions:**

As a measure, frailty captures the known increase in morbidity associated with SMI and may potentially allow for earlier identification of those who will benefit from targeted interventions.

Severe mental illness (SMI), including schizophrenia, bipolar disorder and severe major depression, affects approximately 5% of the adult population and is associated with increased all-cause mortality, leading to an average of 15 years of potential life lost.^1–4^ This is predominantly due to the high prevalence of physical comorbidities, which is twice as high as in the general population and is significantly increased by adverse effects of medications and further compounded by sedentary lifestyle, poor diet and high rates of psychosocial stressors.^5–9^ Given the multifactorial impacts on morbidity and mortality, it can be challenging to identify which individuals with SMI should be prioritised for specific prevention or early intervention services. The concept of frailty, which can encapsulate biological ageing, comorbidity and psychosocial impairments, holds utility in summarising individual needs and allowing complex comparisons. Frailty is a medical syndrome characterised by age-related changes, across multiple body systems, that increase vulnerability to stressors.^10,11^ As a measure, frailty is increasingly used in a wide variety of medical specialties to prognosticate adverse health outcomes, with several frailty measurement tools being developed, although none specific to psychiatric populations.^11–14^ Emerging evidence highlights a higher prevalence of frailty in those with SMI associated with negative health outcomes and increased mortality, although much of this work has focused exclusively on older adults and has been limited by small numbers.^15–17^ Large cohorts, such as the UK Biobank, have been used to show elevated rates of frailty in adults with common mental disorders, such as depression and anxiety.^10^ A small proportion of the previous UK Biobank study population (around 2.6%) included individuals with bipolar disorder and this subgroup showed the largest difference in frailty from the non-psychiatric comparison group and highest hazard ratio for all-cause mortality, although of note, participants with psychosis were excluded from this work.^10^ The primary aim of the present study was to assess the prevalence of frailty in the UK Biobank sample, comparing those with and without a lifetime history of SMI while adjusting for demographic and sociodemographic factors.

## Method

### Participants

A nested cross-sectional analytical study of participants in the UK Biobank baseline assessment was conducted. The UK Biobank is a prospective, multicentre longitudinal cohort study, where 502 412 people aged between 37 and 73 years were non-randomly recruited by mailed invitations (5.5% response rate) and provided baseline data at 1 of 22 assessment centres across England, Scotland and Wales between 13 March 2006 and 1 October 2010.^18^ The study design has been described in detail elsewhere.^19^ Baseline assessment included the completion of a touchscreen questionnaire, nurse-led interview and physical measurements. Hospital episode statistics for in-patients have been linked to the UK Biobank, detailing hospital admissions, diagnoses and mortality.

Presence of SMI was defined as one of the following ICD-10 codes at baseline from hospital records, based on previous work: schizophrenia spectrum disorders: F20.0–29.0; bipolar affective disorder: F31.1–31.9; and severe depression: F32.1–32.3 and F33.1–33.3.^17^ The groups were not mutually exclusive. The comparison group included all other participants of the UK Biobank without an SMI. Sociodemographic confounders came from the UK Biobank baseline survey and included age, gender, ethnicity, employment status and country of birth.

### Outcomes

Three frailty indices were employed: a frailty index, physical frailty phenotype (PFP) and the Hospital Frailty Risk Score (HFRS), with the first two measures previously modified for UK Biobank participants.^20,21^

#### Frailty index

The frailty index used is a 49-item scale calculated by the total number of deficits present divided by total potential deficits and has established cut-offs: relatively fit (≤0.03); less fit (>0.03–0.1); least fit (>0.1–0.21); frail (>0.21); and most frail (≥0.45).^21,22^ These deficits include physical health disorders such as diabetes, asthma and cancer, as well as sensory deficits, pain, measures of well-being and infirmity (Supplementary material A available at https://doi.org/10.1192/bjo.2023.580). Participants with ten or more missing variables were excluded, consistent with previous research.^21^

#### Physical frailty phenotype

The 5-item PFP operationalised with UK Biobank data is calculated based on the presence of weight loss (intended or not) in the past year, exhaustion in the past 2 weeks, low physical activity in the past 4 weeks, grip strength and slow usual walking pace.^23^ Answers were transformed into binary outcomes (Supplementary material B). The PFP categorises participants as: not frail (meeting no criteria); pre-frail (meeting 1 or 2 criteria); and frail (meeting 3–5 criteria). Participants missing one or more variables were excluded.

#### Hospital Frailty Risk Score

The HFRS is a 109-item summary score based on electronic hospital record ICD-10 codes, recorded during a person's hospital admission, that are weighted based on their association with frailty.^24^ It categorises frailty based on the number and weighting of HFRS items present: low risk (<5); intermediate risk (5–15); and high risk (>15) (Supplementary material C). Participants without a recorded hospital admission during the follow-up period were coded as 0, following previous studies using the HFRS.^23^

### Statistical analysis

All statistical analysis and data visualisations were done in Stata 17.0 for Windows^25^ using the user-written command ‘sta Version 1.1.8’.^26^

To examine the distribution of the data across age, considered the key confounder, a stratified analysis was chosen over regression modelling as an initial approach to data analysis. The ratio of frailty prevalence between individuals with each SMI and those without SMI was compared for each of the three frailty measures. Confidence intervals were constructed using the modified Wald method.^27^

As a sensitivity analysis, binary logistic regressions with the frailty index as the dependent variable dichotomised by whether participants were frail (>0.21) or not (≤0.21) were conducted to adjust for multiple variables simultaneously. The assumptions of these regressions were tested with multiple approaches. Two-way tables were used to check that no more than 20% of minimum expected cell frequencies were under five for all combinations of categorical variables. Generalised additive models (GAM) using the Stata module ‘GAM’^28^ examined whether age had logit linearity. Variance inflation factors (VIF) for individual variables and the mean VIF for all variables were inspected for signs of multicollinearity using the ‘collin’^29^ module. Multivariate outliers were detected using the blocked adaptive computationally efficient outlier nominators (BACON) algorithm,^30^ with the 15th percentile of the χ^2^ distribution used as a threshold to distinguish outliers from non-outliers using the ‘bacon’^31^ module in Stata. Confounding bias was addressed through binary logistic regression and recall, and misclassification bias was minimised by using ICD-10 diagnoses of SMI from hospital episode statistics for the HFRS.

### Ethics

The authors assert that all procedures contributing to this work comply with the ethical standards of the relevant national and institutional committees on human experimentation and with the Helsinki Declaration of 1975, as revised in 2008. All procedures involving human subjects were approved by North West Haydock Research Ethics Committee (Reference 16/NW/0274, 13 May 2016) and this research was conducted under application number 81605. All participants in the UK Biobank gave written informed consent for data collection, record linkage and use of data in future research.

## Results

Of the 502 412 participants in the UK Biobank with a baseline assessment, a frailty index could not be calculated for 0.46% of the total sample and a PFP could not be calculated for 0.64%. Regarding the HFRS, a value of 0 was assigned to people without recorded hospital admissions by the end of the baseline assessment (*n* = 168 613, 34%). The demographics and sociodemographic variables for those for whom a frailty index could be calculated are presented in Table 1. There were 2137 individuals with SMI, of whom 251 were coded as having severe depression as well as either schizophrenia spectrum disorders or bipolar affective disorder.

**Table 1:** Demographic characteristics for participants with a frailty index

	SSD	BPAD	Dep	SMI^a^	No SMI
Participants, *n*	885	730	773	2137	497 983
Age when attended assessment centre, years: mean (s.d.)	53.8 (8.17)	55.1 (7.88)	55.5 (8.28)	54.8 (8.17)	56.5 (8.09)
Gender					
Women, *n* (%)	387 (44)	438 (60)	465 (60)	1139 (53)	271 029 (54)
Average total household income before tax, *n* (%)					
<£18 000	454 (70)	328 (54)	309 (50)	961 (57)	95 866 (23)
£18 000–£30 999	117 (18)	129 (21)	158 (25)	364 (22)	107 670 (25)
£31 000-£51 999	49 (8)	83 (14)	98 (16)	212 (13)	110 457 (26)
£52 000–£100 000	27 (4)	52 (9)	55 (9)	125 (7)	86 077 (20)
>£100 000	5 (1)	11 (2)	4 (1)	17 (1)	22 904 (5)
Country of birth, *n* (%)					
England	678 (77)	603 (83)	639 (83)	1727 (81)	387 873 (78)
Wales	48 (5)	24 (3)	43 (6)	99 (5)	21 925 (4)
Scotland	17 (2)	17 (2)	20 (3)	48 (2)	40 036 (8)
Northern Ireland	8 (1)	5 (1)	6 (1)	18 (1)	3077 (1)
Republic of Ireland	22 (2)	14 (2)	7 (1)	38 (2)	4895 (1)
Elsewhere	110 (12)	66 (9)	58 (8)	204 (10)	39 536 (8)
Ethnic background. *n* (%)					
White	768 (87)	680 (94)	732 (95)	1950 (92)	469 925 (95)
Asian or Asian British	23 (3)	8 (1)	12 (2)	39 (2)	9615 (2)
Black or Black British	51 (6)	14 (2)	14 (2)	73 (3)	7871 (2)
Mixed	17 (2)	12 (2)	5 (1)	28 (1)	2920 (1)
Chinese	2 (0)	1 (0)	1 (0)	3 (0)	1544 (0)
Other ethnic group	18 (2)	12 (2)	9 (1)	35 (2)	4477 (1)
Employment status, *n* (%)^b^					
In paid employment or self-employed	165 (19)	195 (27)	226 (29)	542 (26)	286 091 (58)
Retired	240 (27)	233 (32)	272 (35)	672 (32)	176 439 (36)
Looking after home and/or family	44 (5)	52 (7)	28 (4)	110 (5)	25 467 (5)
Unable to work because of sickness or disability	420 (48)	263 (36)	256 (33)	820 (39)	19 357 (4)
Unemployed	67 (8)	32 (4)	26 (3)	110 (5)	9315 (2)
Doing unpaid or voluntary work	75 (9)	69 (10)	49 (6)	167 (8)	18 193 (4)
Full or part-time student	22 (3)	10 (1)	16 (2)	45 (2)	4490 (1)

SSD, ICD-10 schizophrenia, schizotypal and delusional disorders; BPAD, ICD-10 bipolar affective disorders; Dep, ICD-10 severe depression; SMI, severe mental illness.

a. The three individual SMI groups do not total 2137 as 251 participants from these groups had another comorbid SMI.

b. Employment status does not sum to 100.0% as one participant could have multiple responses.

**Fig. 1:**
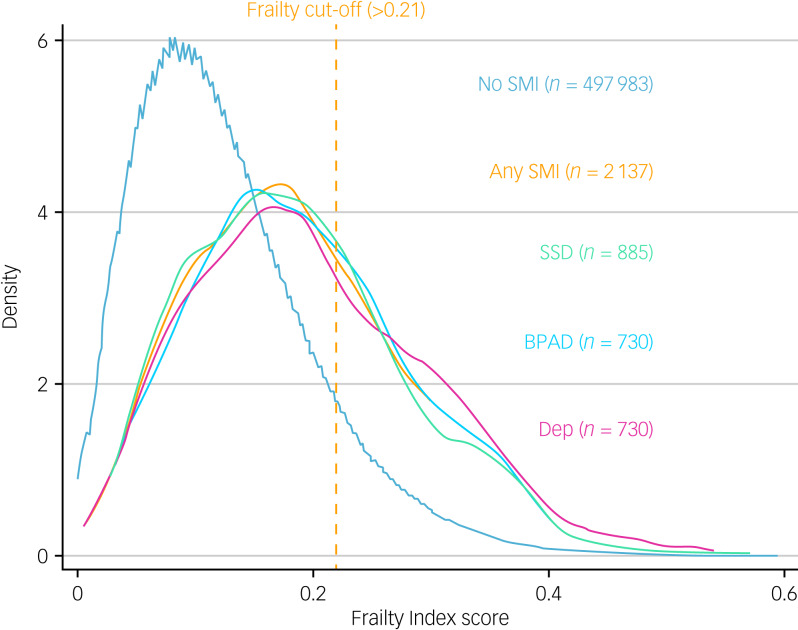
Kernel density plot of the frailty index comparing severe mental illness. SMI, severe mental illness; SSD, ICD-10 schizophrenia, schizotypal and delusional disorders; BPAD, ICD-10 bipolar affective disorders; Dep, ICD-10 severe depression.

The prevalence of frailty in both participants with and without SMI was highest using the frailty index, followed by the PFP and the HFRS (Table 2). Compared with the total cohort, which had a prevalence of frailty using the frailty index of 10.8% (95% CI 10.8–10.9), those with SMI had 3.5 times higher prevalence of frailty. The prevalence ratio was 4–4.7 times higher using the PFP and 17–23 times higher using the HFRS, noting that the prevalence of frailty was low in both the SMI and non-SMI groups when employing the HFRS. In a sensitivity analysis that removed people without SMI who had no hospital admissions from the comparison group, prevalence ratios attenuated to range from 11 to 15 (Supplementary material D). The distribution of frailty in those with schizophrenia spectrum disorders, bipolar affective disorder and severe depression was similar across the three groups (Fig. 1).

**Table 2:** Prevalence of frailty in participants with severe mental illness in the UK Biobank, using three measures

	Frail	Non-frail	Prevalence, % (95% CI)	Prevalence ratio (95% CI)	*P*
Frailty index					
SSD	291	594	32.88 (29.87–36.05)	3.08 (2.80–3.38)	<0.0005
BPAD	260	470	35.62 (32.22–39.16)	3.33 (3.02–3.67)	<0.0005
Dep	285	488	36.87 (33.54–40.33)	3.45 (3.14–3.78)	<0.0005
SMI	729	1408	34.11 (32.13–36.15)	3.19 (3.01–3.39)	<0.0005
No SMI	53 235	444 748	10.69 (10.60–10.78)	n.a.	n.a.
Physical frailty phenotype					
SSD	158	733	17.73 (15.28–20.40)	4.86 (4.22–5.61)	<0.0005
BPAD	107	622	14.68 (12.19–17.46)	4.03 (3.38–4.80)	<0.0005
Dep	113	656	14.69 (12.27–17.40)	4.03 (3.40–4.78)	<0.0005
SMI	328	1809	15.35 (13.85–16.95)	4.21 (3.81–4.66)	<0.0005
No SMI	18 120	478 963	3.65 (3.59–3.70)	n.a.	n.a.
Hospital Frailty Risk Score					
SSD	41	873	4.49 (3.24–6.04)	21.87 (16.12–29.68)	<0.0005
BPAD	26	718	3.50 (2.29–5.08)	17.04 (11.62–24.98)	<0.0005
Dep	37	745	4.73 (3.35–6.46)	23.07 (16.74–31.78)	<0.0005
SMI	82	2099	3.76 (3.00–4.65)	18.33 (14.70–22.86)	<0.0005
No SMI	1026	499 205	0.20 (0.19–0.22)	n.a.	n.a.

SSD, ICD-10 schizophrenia, schizotypal and delusional disorders; BPAD, ICD-10 bipolar affective disorders; Dep, ICD-10 severe depression; SMI, severe mental illness; Prevalence, percentage of participants with frailty compared with total sample; Prevalence ratio: ratio of frailty in people with each SMI to people without SMI; n.a., not applicable.

**Fig. 2:**
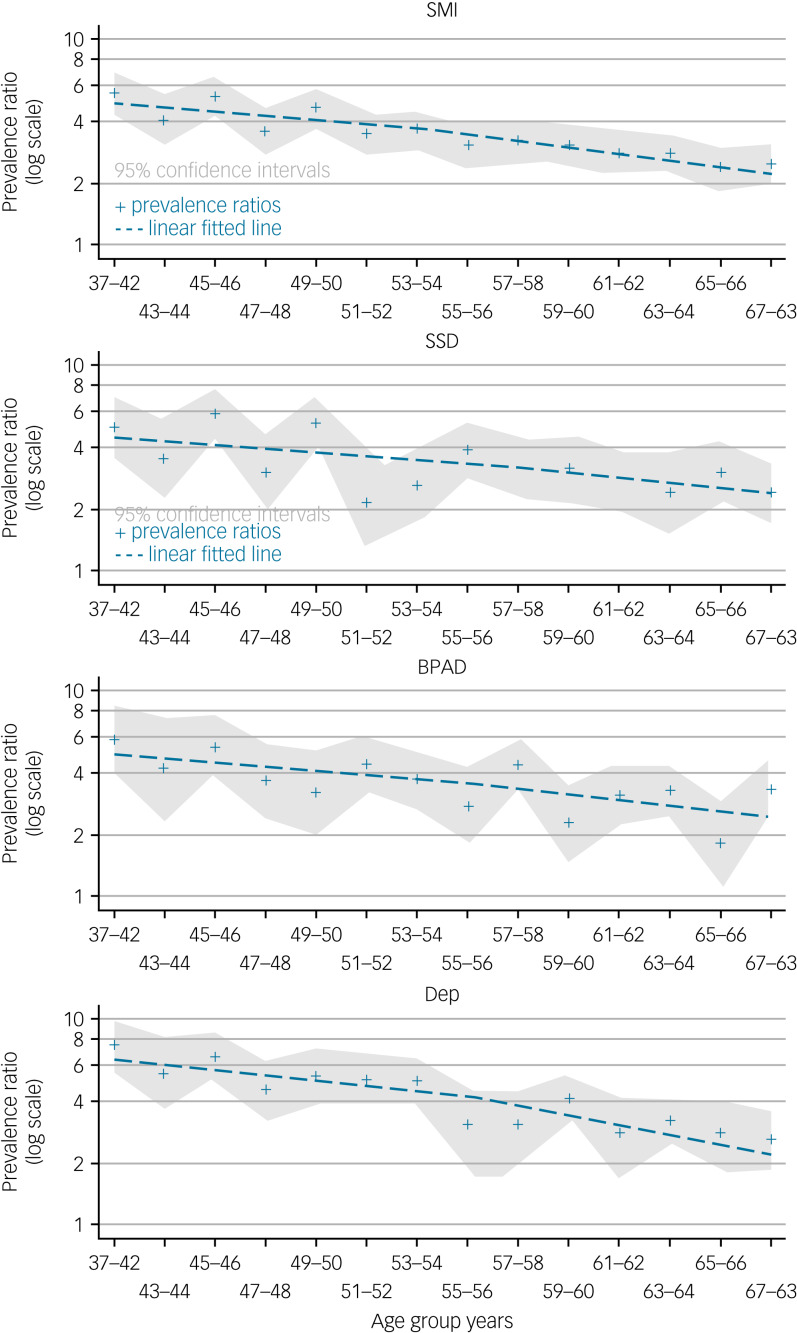
Prevalence ratio with age for severe mental illness using the frailty index. SMI, severe mental illness; SSD, ICD-10 schizophrenia, schizotypal and delusional disorders; BPAD, ICD-10 bipolar affective disorders; Dep, ICD-10 severe depression.

Using the frailty index, the difference in frailty prevalence between participants with SMI and those without SMI, prevalence ratio, is highest in the youngest age group. The difference in frailty prevalence decreases with age for each group of disorders, as well as for all SMI (Fig. 2, Supplementary material E). The reduction of the prevalence ratio, considered to be the narrowing of the prevalence difference between each SMI and no SMI with age, is less in participants with bipolar affective disorder and schizophrenia spectrum disorders compared with severe depression (Fig. 2, Supplementary material E).

Again using the frailty index, frailty was higher in women compared with men in all subgroups, and this difference was greatest in those with bipolar affective disorder, followed by schizophrenia spectrum disorders (Supplementary material F).

Using binary logistic regression to create an adjusted model for age, gender, ethnicity and country of birth (Supplementary material G), the prevalence ratio for each SMI compared with no SMI was elevated, with the greatest change for participants with schizophrenia spectrum disorders (Supplementary materials H, I). When looking at confounders overall, most variables were associated with frailty (Supplementary materials J, K).

## Discussion

This study demonstrates a 3–18 times increase in frailty among people with SMI compared with controls in over 500 000 UK Biobank participants. This is the largest study of frailty in people with SMI and these findings corroborate and enhance those of smaller studies^15–17,32^ and of studies of people with common mental illness^10^ as well as reflecting the known increase of physical health comorbidity in SMI.^5^ Similar to those without SMI, the prevalence of frailty was greater in women and increased with age. In contrast to many studies where associations attenuate after adjusting for confounding variables, the associations here increased slightly, which was probably attributable to people with SMI being on average younger than those without SMI. There was no major confounding in the variables identified from the multivariable analysis.

The prevalence of frailty in SMI was higher than that previously demonstrated in people with depression, anxiety and bipolar affective disorder, also utilising UK Biobank data.^10^ Mutz et al identified participants through a variety of measures, including self-reported hypomania or depression, and excluded all cases with psychosis.^10^ In comparison, this study focused on SMI, which tends to have psychotic symptoms, and utilised only hospital records, resulting in 4 times fewer participants with bipolar affective disorder and 99 times fewer participants with depression. The prevalence of frailty remained higher in those with SMI compared with those without, even in older age groups and especially for those with bipolar affective disorder and schizophrenia spectrum disorders. The significant effects from these severe chronic disorders that continue to occur with age may be related to higher rates of recurrence and the metabolic side-effects of psychotropics.^5^ They may also relate to the cumulative influence of poor health contributors, more common in SMI, such as obesity, smoking, substance use and early life stressors.^5,33^ Of note, there was a difference in sociodemographic factors known to affect development of chronic health conditions (such as employment status and household income) between those with SMI and no SMI; however, these factors could not be adjusted for, as confounder status could not be confirmed in a cross-sectional analysis.

An increased prevalence of frailty is an important finding as it has been shown to be a predictor of hospital admissions, health complications, disability and mortality.^10,34^ Clegg et al demonstrated that a 10% higher baseline in frailty was associated with higher risk of death, especially in those of younger age, which may be especially relevant for the high proportion of younger frail people with SMI.^34^ Frailty has also been demonstrated to be associated with higher levels of cognitive impairment, independent of age, potentially an added concern for the known cognitive deficits associated with SMI.^35–37^ Although frailty interventions have been found to be effective in populations with various medical conditions,^38^ there have been limited frailty intervention studies in those with SMI.^39^ Frailty interventions frequently combine exercise, nutrition, medical and pharmacological review, the importance of which are all well appreciated by those caring for people with SMI. Where the concept of frailty in the context of people with SMI, when measured by a tool such as the frailty index, may be particularly beneficial is to capture a more comprehensive account of an individual's functioning and health. By combining interactive effects of biological ageing, comorbidity and psychosocial impairments, this may facilitate a better understanding of an individual's risk of future adverse health outcomes and, importantly, of their corresponding needs. Such a global assessment of an individual may therefore assist in identifying those who may benefit from targeted early intervention strategies, including psychoeducation, lifestyle behaviour support and self-management. Further, frailty assessment may also inform an individual's treatment plan by pinpointing the specific multidisciplinary treatments and interventions required, while advocating for holistic and person-centred care.

### Limitations

Three frailty assessment tools were utilised in this study and were able to be applied to the majority of the cohort. Although the frailty index and PFP are commonly used tools in SMI studies, the PFP may be less useful in capturing the completeness of deficits in younger frail individuals,^17^ owing to its brevity and non-capture of psychosocial deficits. Additionally, the frailty index may identify the lower- and middle-end distribution of the frailty continuum better, with benefit in identifying those that were at risk of decline and may respond best to intervention.^10^ The HFRS frailty tool gave a much higher prevalence ratio of frailty for those with SMI and for each disorder category compared with the non-SMI group, even when excluding those without SMI who had no hospital admissions; however, the prevalence of frailty in all groups was very low.

There are other limitations that should be considered when interpreting the results of this study. The response rate for recruitment to the UK Biobank, which employed a non-random sampling, was 5.5% and two frailty measures were based on responses from questionaries rather than objectively measured data. The proportion of those with SMI in the UK Biobank cohort is small and there is a selection bias.^40^ This study found that the difference in frailty prevalence in those with compared with those without SMI narrowed especially after the age of 60 years, an occurrence also noted in earlier UK Biobank work on common mental disorders.^10^ Given the volunteer nature of recruitment, those with less severe or stabilised SMI presentations and older-onset SMI may be over-represented. The UK Biobank participants have been shown to be less likely to be obese, smoke or drink alcohol, to have fewer health conditions and live in less socioeconomically deprived areas than the broader UK population.^40^ This may indicate that potentially an even greater frailty could be expected in those with more severe disorders.^32^ However, it should be appreciated that the direction and magnitude of selection bias cannot be determined from this study. Around 12% of the SMI group had more than one SMI diagnosis, and there may have been some misclassification between bipolar affective disorder or schizoaffective disorder and severe depression. This was not included as a confounder in the adjusted model but could be considered in future work. There may also have been cases of SMI not captured by hospital-based ICD-10 coding and misclassified into the control group.

### Future research and implications

Future studies should longitudinally assess frailty, utilise frailty tools that better capture psychological frailty,^14^ consider the effect of SMI and physical health treatments, and review mortality risk associated with frailty in those with SMI. Additionally, pilot feasibility studies of frailty-focused interventions in people with SMI would be critical in informing the development of frailty interventions for people with SMI who are frail.^39^

Regardless of how frailty in operationalised or when it is assessed, the prevalence of frailty appears to be significantly higher in people with SMI than in those without, and it remains elevated throughout life. Frailty can be a more holistic measure, capturing a wide range of deficits and placing an individual on a continuum that can be monitored over time and compared against general population norms. Its clinical utility is in enabling early identification of comorbidity risk among people with SMI and informing the selection of multidisciplinary, person-centred intervention strategies.

## Supporting information

Warren et al. supplementary material 1 Warren et al. supplementary material

Warren et al. supplementary material 2 Warren et al. supplementary material

## Data Availability

Data to support the findings are available in the UK Biobank and further analyses of the data can be found in the supplementary material.
