# Supplementary material for: Prevalence of frailty in severe mental illness: findings from the UK Biobank
Source: BJPsych Open. 2023 Oct 12;9(6):e185. doi: 10.1192/bjo.2023.580 (PMC10594184; doi:10.1192/bjo.2023.580)
Supplement: Warren et al. supplementary material 1 — Warren et al. supplementary material [file S205647242300580Xsup001.docx]

**Supplementary Material A:**

**Table: Questionnaire items from the baseline UK Biobank assessment used to compose the Frailty Index**

| **Type of deficit** | **Item** | **Trait** | **Categories** | **Coding in the FI item** |  |
| --- | --- | --- | --- | --- | --- |
|  |  |  |  |  |  |
| *Sensory* | 1 | Glaucoma * | no, yes | Categorised 0/1 |  |
|  | 2 | Cataracts * | no, yes | Categorised 0/1 |  |
|  | 3 | Hearing difficulty | no, yes, completely deaf | Categorised 0/1 |  |
|  |  |  |  | (Combined yes/deaf groups as 1) |  |
| *Cranial* | 4 | Migraine * | no, yes | Categorised 0/1 |  |
|  | 5 | Dental problems | ulcers, painful gums, bleeding gums, loose teeth, toothache, dentures | Categorised 0/1 for none vs. any |  |
| *Mental wellbeing* | 6 | Self-rated health | excellent, good, fair, poor | 0 – excellent; 0.25 – good; 0.5 – fair,  1 – poor |  |
|  | 7 | Fatigue: frequency of tiredness/lethargy in last two weeks | not at all, several days, more than half, nearly every day | 0, 0.25, 0.5, 1, respectively |  |
|  | 8 | Sleep: experience of sleeplessness/insomnia | never/rarely, sometimes, usually | Categorised 0, 0.5, 1, respectively |  |
|  | 9 | Depressed feelings: frequency in last two weeks | not at all, several days, more than half, nearly every day | 0 – not at all, 0.5 – several days, 0.75 – more than half, 1 – nearly every day |  |
|  |  |  |  |  |  |
|  | 10 | Self-described nervous personality | no, yes | Categorised 0/1 |  |
|  | 11 | Severe anxiety/ panic attacks * | no, yes | Categorised 0/1 |  |
|  | 12 | Common to feel loneliness | no, yes | Categorised 0/1 |  |
|  | 13 | Sense of misery (ever/never) | no, yes | Categorised 0/1 |  |
| *Infirmity* | 14 | Infirmity: long-standing illness or disability | no, yes | Categorised 0/1 |  |
|  | 15 | Falls in last year | categorical: no falls, one fall, more than one | 0, 0.5, 1, respectively |  |
|  | 16 | Fractures/broken bones in last five years | no, yes | Categorised 0/1 |  |
| *Cardiometabolic* | 17 | Diabetes * | no, yes | Categorised 0/1 |  |
|  | 18 | Myocardial infarction * | no, yes | Categorised 0/1 |  |
|  | 19 | Angina * | no, yes | Categorised 0/1 |  |
|  | 20 | Stroke * | no, yes | Categorised 0/1 |  |
|  | 21 | High blood pressure * | no, yes | Categorised 0/1 |  |
|  | 22 | Hypothyroidism * | no, yes | Categorised 0/1 |  |
|  | 23 | Deep-vein thrombosis * | no, yes | Categorised 0/1 |  |
|  | 24 | High cholesterol * | no, yes | Categorised 0/1 |  |
| *Respiratory* | 25 | Breathing: wheeze in last year | no, yes | Categorised 0/1 |  |
|  | 26 | Pneumonia * | no, yes | Categorised 0/1 |  |
|  | 27 | Chronic bronchitis/emphysema * | no, yes | Categorised 0/1 |  |
|  | 28 | Asthma * | no, yes | Categorised 0/1 |  |
| *Musculoskeletal* | 29 | Rheumatoid arthritis * | no, yes | Categorised 0/1 |  |
|  | 30 | Osteoarthritis * | no, yes | Categorised 0/1 |  |
|  | 31 | Gout * | no, yes | Categorised 0/1 |  |
|  | 32 | Osteoporosis * | no, yes | Categorised 0/1 |  |
| *Immunological* | 33 | Hayfever, allergic rhinitis or eczema * | no, yes | Categorised 0/1 |  |
|  | 34 | Psoriasis * | no, yes | Categorised 0/1 |  |
| *Cancer* | 35 | Any cancer diagnosis * | no, yes | Categorised 0/1 |  |
|  | 36 | Multiple cancers diagnosed (number reported) | Range from 0 to 6 | 0 - no cancer or single cancer, 1 - multiple cancers |  |
| *Pain* | 37 | Chest pain | no, yes | Categorised 0/1 |  |
|  | 38 | Head and/or neck pain | no, yes (combining responses to pain in head and neck/shoulders) | Categorised 0/1 |  |
|  | 39 | Back pain | no, yes | Categorised 0/1 |  |
|  | 40 | Stomach/abdominal pain | no, yes | Categorised 0/1 |  |
|  | 41 | Hip pain | no, yes | Categorised 0/1 |  |
|  | 42 | Knee pain | no, yes | Categorised 0/1 |  |
|  | 43 | Whole-body pain | no, yes | Categorised 0/1 |  |
|  | 44 | Facial pain | no, yes | Categorised 0/1 |  |
|  | 45 | Sciatica * | no, yes | Categorised 0/1 |  |
| *Gastrointestinal* | 46 | Gastric reflux * | no, yes | Categorised 0/1 |  |
|  | 47 | Hiatus hernia * | no, yes | Categorised 0/1 |  |
|  | 48 | Gall stones * | no, yes | Categorised 0/1 |  |
|  | 49 | Diverticulitis * | no, yes | Categorised 0/1 |  |

† Numbers indicate data availability in the whole cohort (from total *N* = 502,412)

* Participants reported medically diagnosed conditions for these items

**Supplementary Material B:**

**Table: Questionnaire items from the baseline UK Biobank assessment used to compose the Physical Frailty Phenotype**

| **Type of deficit** | **Question** | **Response** | **Categories** | **Coding in the PFP item** |  |
| --- | --- | --- | --- | --- | --- |
| Weight loss | "Compared with one year ago, has your weight changed?" | \| **Coding** \| **Meaning** \| \| --- \| --- \| \| 0 \| No - weigh about the same \| \| 2 \| Yes - gained weight \| \| 3 \| Yes - lost weight \| \| -1 \| Do not know \| \| -3 \| Prefer not to answer \| | Other, lost weight (code 3) | Categorised 0/1 |  |
| Exhaustion | "Over the past two weeks, how often have you felt tired or had little energy?" | \| **Coding** \| **Meaning** \| \| --- \| --- \| \| 1 \| Not at all \| \| 2 \| Several days \| \| 3 \| More than half the days \| \| 4 \| Nearly every day \| \| -1 \| Do not know \| \| -3 \| Prefer not to answer \| | No, yes (codes 3 and 4) | Categorised 0/1 |  |
| Physical activity | "In the last 4 weeks did you spend any time doing the following? (You can select more than one answer)", "How many times in the last 4 weeks did you do light DIY?" |  | No, yes | Categorised 0/1 |  |
| Walking speed | "How would you describe your usual walking pace?" | **Coding Meaning**  1 Slow pace  2 Steady average pace  3 Brisk pace  -7 None of the above  -3Prefer not to answer | No, yes (code 1) | Categorised 0/1 |  |
| Grip strength |  |  | No, yes | Categorised 0/1 |  |

**Supplementary Material C:**

**HFRS Coding: ICD-10 Code, Diagnosis, Points Awarded**

F00 Dementia in Alzheimer's disease 7·1

G81 Hemiplegia 4·4

G30 Alzheimer's disease 4·0

I69 Sequelae of cerebrovascular disease (secondary codes) 3·7

R29 Other symptoms and signs involving the nervous and musculoskeletal systems (R29·6 Tendency to fall) 3·6

N39 Other disorders of urinary system (includes urinary tract infection and urinary incontinence)3·2

F05 Delirium, not induced by alcohol and other psychoactive substances 3·2

W19 Unspecified fall 3·2

S00 Superficial injury of head 3·2

R31 Unspecified haematuria 3·0

B96 Other bacterial agents as the cause of diseases classified to other chapters (secondary code) 2·9

R41 Other symptoms and signs involving cognitive functions and awareness 2·7

R26 Abnormalities of gait and mobility 2·6

I67 Other cerebrovascular diseases 2·6

R56 Convulsions, not elsewhere classified 2·6

R40 Somnolence, stupor and coma 2·5

T83 Complications of genitourinary prosthetic devices,implants and grafts2·4

S06 Intracranial injury 2·4

S42 Fracture of shoulder and upper arm 2·3

E87 Other disorders of fluid, electrolyte and acid-base balance 2·3

M25 Other joint disorders, not elsewhere classified 2·3

*E86 Volume depletion 2·3*

*R54 Senility 2·2*

*Z50 Care involving use of rehabilitation procedures 2·1*

*F03 Unspecified dementia 2·1*

*W18 Other fall on same level 2·1*

*Z75 Problems related to medical facilities and other healthcare2·0*

*F01 Vascular dementia 2·0*

*S80 Superficial injury of lower leg 2·0*

*L03 Cellulitis 2·0*

*H54 Blindness and low vision 1·9*

*E53 Deficiency of other B group vitamins 1·9*

*Z60 Problems related to social environment 1·8*

*G20 Parkinson's disease 1·8*

*R55 Syncope and collapse 1·8*

S22 Fracture of rib(s), sternum and thoracic spine 1·8

K59 Other functional intestinal disorders 1·8

N17 Acute renal failure 1·8

L89 Decubitus ulcer 1·7

Z22 Carrier of infectious disease 1·7

B95 Streptococcus and staphylococcus as the cause of diseases classified to other chapters1·7

L97 Ulcer of lower limb, not elsewhere classified 1·6

R44 Other symptoms and signs involving general sensations and perceptions 1·6

K26 Duodenal ulcer 1·6

I95 Hypotension 1·6

N19 Unspecified renal failure 1·6

A41 Other septicaemia 1·6

Z87 Personal history of other diseases and conditions 1·5

J96 Respiratory failure, not elsewhere classified 1·5

X59 Exposure to unspecified factor 1·5

M19 Other arthrosis 1·5

G40 Epilepsy 1·5

M81 Osteoporosis without pathological fracture 1·4

S72 Fracture of femur 1·4

S32 Fracture of lumbar spine and pelvis 1·4

E16 Other disorders of pancreatic internal secretion 1·4

R94 Abnormal results of function studies 1·4

N18 Chronic renal failure 1·4

R33 Retention of urine 1·3

R69 Unknown and unspecified causes of morbidity 1·3

N28 Other disorders of kidney and ureter, not elsewhere classified1·3

R32 Unspecified urinary incontinence 1·2

G31 Other degenerative diseases of nervous system, not elsewhere classified1·2

Y95 Nosocomial condition 1·2

S09 Other and unspecified injuries of head 1·2

R45 Symptoms and signs involving emotional state 1·2

G45 Transient cerebral ischaemic attacks and related syndromes 1·2

Z74 Problems related to care-provider dependency 1·1

M79 Other soft tissue disorders, not elsewhere classified 1·1

W06 Fall involving bed 1·1

S01 Open wound of head 1·1

A04 Other bacterial intestinal infections 1·1

A09 Diarrhoea and gastroenteritis of presumed infectiou sorigin 1·1

J18 Pneumonia, organism unspecified 1·1

J69 Pneumonitis due to solids and liquids 1·0

R47 Speech disturbances, not elsewhere classified 1·0

E55 Vitamin D deficiency 1·0

Z93 Artificial opening status 1·0

R02 Gangrene, not elsewhere classified 1·0

R63 Symptoms and signs concerning food and fluid intake 0·9

H91 Other hearing loss 0·9

W10 Fall on and from stairs and steps 0·9

W01 Fall on same level from slipping, tripping and stumbling 0·9

E05 Thyrotoxicosis [hyperthyroidism] 0·9

M41 Scoliosis 0·9

R13 Dysphagia 0·8

Z99 Dependence on enabling machines and devices 0·8

U80 Agent resistant to penicillin and related antibiotics 0·8

M80 Osteoporosis with pathological fracture 0·8

K92 Other diseases of digestive system 0·8

I63 Cerebral Infarction 0·8

N20 Calculus of kidney and ureter 0·7

F10 Mental and behavioural disorders due to use of alcohol 0·7

Y84 Other medical procedures as the cause of abnormal reaction of the patient 0·7

R00 Abnormalities of heart beat 0·7

J22 Unspecified acute lower respiratory infection 0·7

Z73 Problems related to life-management difficulty 0·6

R79 Other abnormal findings of blood chemistry 0·6

Z91 Personal history of risk-factors, not elsewhere classified 0·5

S51 Open wound of forearm 0·5

F32 Depressive episode 0·5

M48 Spinal stenosis (secondary code only) 0·5

E83 Disorders of mineral metabolism 0·4

M15 Polyarthrosis 0·4

D64 Other anaemias 0·4

L08 Other local infections of skin and subcutaneous tissue 0·4

R11 Nausea and vomiting 0·3

K52 Other noninfective gastroenteritis and colitis 0·3

R50 Fever of unknown origin 0·1

**Supplementary Material D:**

**Table 2** 2x2 tables, prevalence of frailty, measures of association and *P* values, HFRS sensitivity analysis, UK Biobank, 2006 to 2010

|  | Frail | Non frail | *N* in the comparison with no SMI  [% of total UK Biobank sample] | Prevalence as a percentage  [95% Exact CIs] | Prevalence difference as a percentage  [95% CIs ^a^] | Prevalence ratio  [95% CIs] | *P* value ^b^ |
| --- | --- | --- | --- | --- | --- | --- | --- |
| Hospital Frailty Risk Score – Those not hospitalised coded as zero | | | | | | | |
| SSD | 41 | 873 | 501 145 [99.7] | 4.5  [3.2 to 6.0] | 4.3  [3.1 to 5.8] | 22  [16 to 30] | < 0.0005 |
| BPAD | 26 | 718 | 500 975 [99.7] | 3.5  [2.3 to 5.1] | 3.3  [2.2 to 4.9] | 17  [11 to 25] | < 0.0005 |
| Dep | 37 | 745 | 501 013 [99.7] | 4.7  [3.4 to 6.5] | 4.5  [3.2 to 6.3] | 23  [17 to 32] | < 0.0005 |
| SMI | 82 | 2 099 | 502 412 [100.0] | 3.8  [3.0 to 4.6] | 3.6  [2.8 to 4.4] | 18  [15 to 23] | < 0.0005 |
| No SMI  (Ref group) | 1 026 | 499 205 | na | 0.20  [0.19 to 0.22] | na | na | na |
| Hospital Frailty Risk Score – Only those hospitalised included | | | | | | | |
| SSD | 41 | 873 | 332 532  [66.2] | 4.5  [3.2 to 6.0] | 4.2  [3.0 to 5.7] | 14  [11 to 20] | < 0.0005 |
| BPAD | 26 | 718 | 332 362  [66.2] | 3.5  [2.3 to 5.1] | 3.2  [2.1 to 4.8] | 11  [7.7 to 17] | < 0.0005 |
| Dep | 37 | 745 | 332 400  [66.2] | 4.7  [3.4 to 6.5] | 4.4  [3.1 to 6.1] | 15  [11 to 21] | < 0.0005 |
| SMI | 82 | 2 099 | 333 799  [66.4] | 3.8  [3.0 to 4.6] | 3.45  [2.7 to 4.3] | 12  [10 to 15] | < 0.0005 |
| No SMI  (Ref group) | 1 026 | 330 592 | na | 0.31  [0.29 to 0.33] | na | na | na |

BPAD = ICD-10 Bipolar affective disorders; na = not applicable; Dep = ICD-10 severe depression; SSD = ICD-10 schizophrenia, schizotypal and delusional disorders ^a^ The 95% confidence intervals for the prevalence difference use the Newcombe formula. ^b^ Both 1-sided and 2-sided confidence intervals. We report an inequality (i.e., ‘<’) as Stata reported p = 0.0000. The P value is a continuous measure indicating how compatible (larger P values) or incompatible (smaller P values) the data are with the null hypothesis of no difference in the prevalence of frailty between each SMI and people without SMI in the UK population (excluding Northern Ireland), assuming that random sampling has occurred.

| **Supplementary Material E:**  **Table:** Data underlying Figure 2 for people with schizophrenia, schizotypal or delusional disorder vs non-SMI | | | | |
| --- | --- | --- | --- | --- |
| **Age group** | **SMI status** | **Frail** | **Not frail** | **TOTAL** |
| 37 to 42 | SSD | 25 | 53 | 78 |
|  | No SMI | 1 777 | 25 554 | 27 331 |
|  | TOTAL | 1 802 | 25 607 | 27 409 |
| 43 to 44 | SSD | 16 | 49 | 65 |
|  | No SMI | 1 673 | 22 118 | 23 791 |
|  | TOTAL | 1 689 | 22 167 | 23 856 |
| 45 to 46 | SSD | 29 | 40 | 69 |
|  | No SMI | 1 843 | 23 221 | 25 064 |
|  | TOTAL | 1 872 | 23 261 | 25 133 |
| 47 to 48 | SSD | 16 | 51 | 67 |
|  | No SMI | 2 085 | 24 142 | 26 227 |
|  | TOTAL | 2 101 | 24 193 | 26 294 |
| 49 to 50 | SSD | 29 | 38 | 67 |
|  | No SMI | 2 324 | 25 829 | 28 153 |
|  | TOTAL | 2 353 | 25 867 | 28 220 |
| 51 to 52 | SSD | 13 | 51 | 64 |
|  | No SMI | 2 828 | 27 108 | 29 936 |
|  | TOTAL | 2 841 | 27 159 | 30 000 |
| 53 to 54 | SSD | 18 | 50 | 68 |
|  | No SMI | 3 216 | 28 285 | 31 501 |
|  | TOTAL | 3 234 | 28 335 | 31 569 |
| 55 to 56 | SSD | 24 | 34 | 58 |
|  | No SMI | 3 622 | 30 307 | 33 929 |
|  | TOTAL | 3 646 | 30 341 | 33 987 |
| 57 to 58 | SSD | 25 | 45 | 70 |
|  | No SMI | 4 087 | 32 255 | 36 342 |
|  | TOTAL | 4 112 | 32 300 | 36 412 |
| 59 to 60 | SSD | 18 | 33 | 51 |
|  | No SMI | 4 954 | 39 065 | 44 019 |
|  | TOTAL | 4 972 | 39 098 | 44 070 |
| 61 to 62 | SSD | 22 | 47 | 69 |
|  | No SMI | 5 891 | 45 108 | 50 999 |
|  | TOTAL | 5 913 | 45 155 | 51 068 |
| 63 to 64 | SSD | 14 | 33 | 47 |
|  | No SMI | 5 645 | 39 753 | 45 398 |
|  | TOTAL | 5 659 | 39 786 | 45 445 |
| 65 to 66 | SSD | 21 | 32 | 53 |
|  | No SMI | 5 496 | 36 521 | 42 017 |
|  | TOTAL | 5 517 | 36 553 | 42 070 |
| 67 to 73 | SSD | 21 | 38 | 59 |
|  | No SMI | 7 794 | 45 482 | 53 276 |
|  | TOTAL | 7 815 | 45 520 | 53 335 |
| OVERALL | SSD | 291 | 594 | 885 |
|  | No SMI | 53 235 | 444 748 | 497 983 |
|  | TOTAL | 53 526 | 445 342 | 498 868 |

| **Table:** Data underlying Figure 2 for people with bipolar affective disorder vs non-SMI | | | | |
| --- | --- | --- | --- | --- |
| **Age group** | **SMI status** | **Frail** | **Not frail** | **TOTAL** |
| 37 to 42 | BPAD | 19 | 31 | 50 |
|  | No SMI | 1 777 | 25 554 | 27 331 |
|  | TOTAL | 1 796 | 25 585 | 27 381 |
| 43 to 44 | BPAD | 8 | 19 | 27 |
|  | No SMI | 1 673 | 22 118 | 23 791 |
|  | TOTAL | 1 681 | 22 137 | 23 818 |
| 45 to 46 | BPAD | 20 | 31 | 51 |
|  | No SMI | 1 843 | 23 221 | 25 064 |
|  | TOTAL | 1 863 | 23 252 | 25 115 |
| 47 to 48 | BPAD | 15 | 36 | 51 |
|  | No SMI | 2 085 | 24 142 | 26 227 |
|  | TOTAL | 2 100 | 24 178 | 26 278 |
| 49 to 50 | BPAD | 13 | 36 | 49 |
|  | No SMI | 2 324 | 25 829 | 28 153 |
|  | TOTAL | 2 337 | 25 865 | 28 202 |
| 51 to 52 | BPAD | 23 | 32 | 55 |
|  | No SMI | 2 828 | 27 108 | 29 936 |
|  | TOTAL | 2 851 | 27 140 | 29 991 |
| 53 to 54 | BPAD | 22 | 36 | 58 |
|  | No SMI | 3 216 | 28 285 | 31 501 |
|  | TOTAL | 3 238 | 28 321 | 31 559 |
| 55 to 56 | BPAD | 15 | 36 | 51 |
|  | No SMI | 3 622 | 30 307 | 33 929 |
|  | TOTAL | 3 637 | 30 343 | 33 980 |
| 57 to 58 | BPAD | 27 | 28 | 55 |
|  | No SMI | 4 087 | 32 255 | 36 342 |
|  | TOTAL | 4 114 | 32 283 | 36 397 |
| 59 to 60 | BPAD | 15 | 43 | 58 |
|  | No SMI | 4 954 | 39 065 | 44 019 |
|  | TOTAL | 4 969 | 39 108 | 44 077 |
| 61 to 62 | BPAD | 21 | 37 | 58 |
|  | No SMI | 5 891 | 45 108 | 50 999 |
|  | TOTAL | 5 912 | 45 145 | 51 057 |
| 63 to 64 | BPAD | 30 | 43 | 73 |
|  | No SMI | 5 645 | 39 753 | 45 398 |
|  | TOTAL | 5 675 | 39 796 | 45 471 |
| 65 to 66 | BPAD | 13 | 42 | 55 |
|  | No SMI | 5 496 | 36 521 | 42 017 |
|  | TOTAL | 5 509 | 36 563 | 42 072 |
| 67 to 73 | BPAD | 19 | 20 | 39 |
|  | No SMI | 7 794 | 45 482 | 53 276 |
|  | TOTAL | 7 813 | 45 502 | 53 315 |
| OVERALL | BPAD | 260 | 470 | 730 |
|  | No SMI | 53 235 | 444 748 | 497 983 |
|  | TOTAL | 53 495 | 445 218 | 498 713 |

| **Table:** Data underlying Figure 2 for people with severe depression vs non-SMI | | | | |
| --- | --- | --- | --- | --- |
| **Age group** | **SMI status** | **Frail** | **Not frail** | **TOTAL** |
| 37 to 42 | Dep | 25 | 29 | 54 |
|  | No SMI | 1 777 | 25 554 | 27 331 |
|  | TOTAL | 1 802 | 25 583 | 27 385 |
| 43 to 44 | Dep | 13 | 24 | 37 |
|  | No SMI | 1 673 | 22 118 | 23 791 |
|  | TOTAL | 1 686 | 22 142 | 23 828 |
| 45 to 46 | Dep | 24 | 28 | 52 |
|  | No SMI | 1 843 | 23 221 | 25 064 |
|  | TOTAL | 1 867 | 23 249 | 25 116 |
| 47 to 48 | Dep | 18 | 38 | 56 |
|  | No SMI | 2 085 | 24 142 | 26 227 |
|  | TOTAL | 2 103 | 24 180 | 26 283 |
| 49 to 50 | Dep | 18 | 27 | 45 |
|  | No SMI | 2 324 | 25 829 | 28 153 |
|  | TOTAL | 2 342 | 25 856 | 28 198 |
| 51 to 52 | Dep | 21 | 27 | 48 |
|  | No SMI | 2 828 | 27 108 | 29 936 |
|  | TOTAL | 2 849 | 27 135 | 29 984 |
| 53 to 54 | Dep | 27 | 31 | 58 |
|  | No SMI | 3 216 | 28 285 | 31 501 |
|  | TOTAL | 3 243 | 28 316 | 31 559 |
| 55 to 56 | Dep | 13 | 33 | 46 |
|  | No SMI | 3 622 | 30 307 | 33 929 |
|  | TOTAL | 3 635 | 30 340 | 33 975 |
| 57 to 58 | Dep | 15 | 35 | 50 |
|  | No SMI | 4 087 | 32 255 | 36 342 |
|  | TOTAL | 4 102 | 32 290 | 36 392 |
| 59 to 60 | Dep | 29 | 41 | 70 |
|  | No SMI | 4 954 | 39 065 | 44 019 |
|  | TOTAL | 4 983 | 39 106 | 44 089 |
| 61 to 62 | Dep | 14 | 37 | 51 |
|  | No SMI | 5 891 | 45 108 | 50 999 |
|  | TOTAL | 5 905 | 45 145 | 51 050 |
| 63 to 64 | Dep | 29 | 55 | 84 |
|  | No SMI | 5 645 | 39 753 | 45 398 |
|  | TOTAL | 5 674 | 39 808 | 45 482 |
| 65 to 66 | Dep | 17 | 37 | 54 |
|  | No SMI | 5 496 | 36 521 | 42 017 |
|  | TOTAL | 5 513 | 36 558 | 42 071 |
| 67 to 73 | Dep | 22 | 46 | 68 |
|  | No SMI | 7 794 | 45 482 | 53 276 |
|  | TOTAL | 7 816 | 45 528 | 53 344 |
| OVERALL | Dep | 285 | 488 | 773 |
|  | No SMI | 53 235 | 444 748 | 497 983 |
|  | TOTAL | 53 520 | 445 236 | 498 756 |

| **Table:** Data underlying Figure2 for people with severe mental illness vs non-SMI | | | | |
| --- | --- | --- | --- | --- |
| **Age group** | **SMI status** | **Frail** | **Not frail** | **TOTAL** |
| 37 to 42 | SMI | 56 | 102 | 158 |
|  | No SMI | 1,777 | 25,554 | 27,331 |
|  | TOTAL | 1,833 | 25,656 | 27,489 |
| 43 to 44 | SMI | 35 | 88 | 123 |
|  | No SMI | 1,673 | 22,118 | 23,791 |
|  | TOTAL | 1,708 | 22,206 | 23,914 |
| 45 to 46 | SMI | 57 | 90 | 147 |
|  | No SMI | 1,843 | 23,221 | 25,064 |
|  | TOTAL | 1,900 | 23,311 | 25,211 |
| 47 to 48 | SMI | 44 | 111 | 155 |
|  | No SMI | 2,085 | 24,142 | 26,227 |
|  | TOTAL | 2,129 | 24,253 | 26,382 |
| 49 to 50 | SMI | 56 | 90 | 146 |
|  | No SMI | 2,324 | 25,829 | 28,153 |
|  | TOTAL | 2,380 | 25,919 | 28,299 |
| 51 to 52 | SMI | 47 | 98 | 145 |
|  | No SMI | 2,828 | 27,108 | 29,936 |
|  | TOTAL | 2,875 | 27,206 | 30,081 |
| 53 to 54 | SMI | 62 | 107 | 169 |
|  | No SMI | 3,216 | 28,285 | 31,501 |
|  | TOTAL | 3,278 | 28,392 | 31,670 |
| 55 to 56 | SMI | 45 | 93 | 138 |
|  | No SMI | 3,622 | 30,307 | 33,929 |
|  | TOTAL | 3,667 | 30,400 | 34,067 |
| 57 to 58 | SMI | 53 | 94 | 147 |
|  | No SMI | 4,087 | 32,255 | 36,342 |
|  | TOTAL | 4,140 | 32,349 | 36,489 |
| 59 to 60 | SMI | 56 | 106 | 162 |
|  | No SMI | 4,954 | 39,065 | 44,019 |
|  | TOTAL | 5,010 | 39,171 | 44,181 |
| 61 to 62 | SMI | 53 | 111 | 164 |
|  | No SMI | 5,891 | 45,108 | 50,999 |
|  | TOTAL | 5,944 | 45,219 | 51,163 |
| 63 to 64 | SMI | 64 | 119 | 183 |
|  | No SMI | 5,645 | 39,753 | 45,398 |
|  | TOTAL | 5,709 | 39,872 | 45,581 |
| 65 to 66 | SMI | 45 | 99 | 144 |
|  | No SMI | 5,496 | 36,521 | 42,017 |
|  | TOTAL | 5,541 | 36,620 | 42,161 |
| 67 to 73 | SMI | 56 | 100 | 156 |
|  | No SMI | 7,794 | 45,482 | 53,276 |
|  | TOTAL | 7,850 | 45,582 | 53,432 |
| OVERALL | SMI | 729 | 1,408 | 2,137 |
|  | No SMI | 53,235 | 444,748 | 497,983 |
|  | TOTAL | 53,964 | 446,156 | 500,120 |

**Supplementary Material F:**

**Figure:** Frailty prevalence in severe mental illness by sex


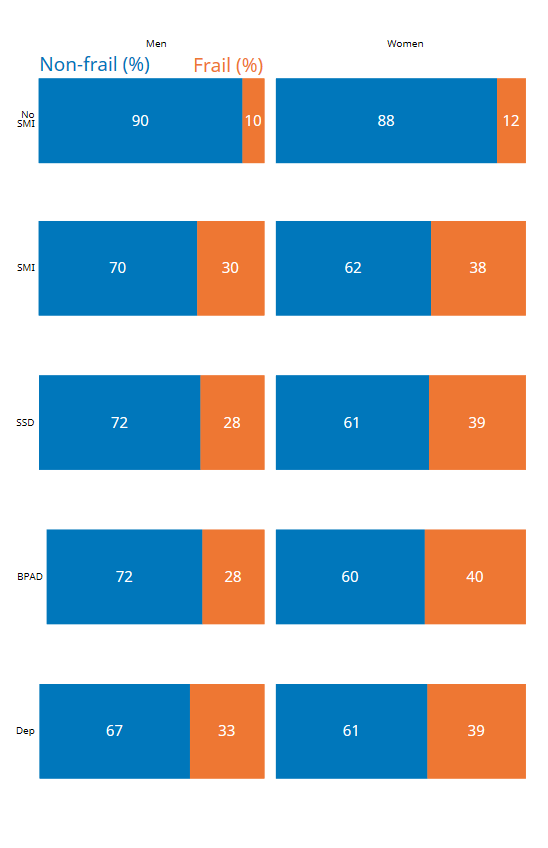


**Supplementary Material G:**

**Table:** Demographic characteristics, by severe mental illness, people with a Frailty Index score included in regressions

|  | **SSD** | **BPAD** | **Dep** | **Any severe mental illness** | | |
| --- | --- | --- | --- | --- | --- | --- |
|  |  |  |  | **Yes** | **No** | |
| Missing data on confounders and thus  excluded from the regressions – *n* [%] | 8 [0.9] | 4 [0.5] | 0 [0.0] | 12 [0.6] | 2 171 [0.4] | |
| Frequency [% of UK Biobank] ^a^ | 877 [0.17] | 726 [0.14] | 773 [0.15] | 2 125 [0.42] | 495 812 [99] | |
| Age |  |  |  |  |  | |
| Mean [SD] | 53.8 [8.18] | 55.2 [7.89] | 55.5 [8.28] | 54.8 [8.18] | 56.6 [8.09] | |
| Sex |  |  |  |  |  | |
| Women - *n* [%] | 383 [44] | 435 [60] | 465 [60] | 1 132 [53] | 270 034 [54] | |
| Country of birth – *n* [%] |  |  |  |  |  |  |
| England | 675 [77] | 601 [83] | 639 [83] | 1 722 [81] | 386 802 [78] | |
| Wales | 48 [5.5] | 24 [3.3] | 43 [5.6] | 99 [4.7] | 21 880 [4.4] | |
| Scotland | 17 [1.9] | 17 [2.3] | 20 [2.6] | 48 [2.3] | 39 959 [8.1] | |
| Northern Ireland | 8 [0.9] | 5 [0.7] | 6 [0.8] | 18 [0.9] | 3 064 [0.6] | |
| Republic of Ireland | 22 [2.5] | 14 [1.9] | 7 [0.9] | 38 [1.8] | 4 883 [1.0] | |
| Elsewhere | 107 [12.2] | 65 [9.0] | 58 [7.5] | 200 [9.4] | 39 224 [7.9] | |
| Ethnic background – *n* [%] |  |  |  |  |  |  |
| White | 767 [87.5] | 680 [94] | 732 [95] | 1 949 [92] | 469 791 [95] | |
| Asian or Asian British or Chinese | 24 [2.7] | 9 [1.2] | 13 [1.7] | 41 [1.9] | 10 916 [2.2] | |
| Black or Black British | 51 [5.8] | 14 [1.9] | 14 [1.8] | 73 [3.4] | 7 792 [1.6] | |
| Mixed | 17 [1.9] | 11 [1.5] | 5 [0.6] | 27 [1.3] | 2 907 [0.6] | |
| Other ethnic group | 18 [2.0] | 12 [1.6] | 9 [1.2] | 35 [1.6] | 4 406 [0.9] | |

^a^ Due to missing data on age, sex, country of birth or ethnic background

# Supplementary Material H:

# Table: Prevalence frailty ratios in participants with SMI compared to no SMI using a model adjusted for age, sex, ethnicity and country of birth

| **Frailty Index** | **Unadjusted model** | | | **Adjusted model** | | | **% change from the unadjusted to the adjusted estimate** |
| --- | --- | --- | --- | --- | --- | --- | --- |
|  | **PR** | **95% CI** | | **PR** | **95% CI** | |  |
|  |  | **Lower limit** | **Upper limit** |  | **Lower limit** | **Upper limit** |  |
| SSD | 3.08 | 2.80 | 3.38 | 3.23 | 2.95 | 3.55 | 4.9 |
| BPAD | 3.33 | 3.02 | 3.67 | 3.39 | 3.08 | 3.73 | 1.8 |
| Dep | 3.45 | 3.14 | 3.78 | 3.51 | 3.20 | 3.84 | 1.7 |
| SMI | 3.19 | 3.01 | 3.39 | 3.29 | 3.10 | 3.49 | 3.1 |

*PR* = prevalence ratio, *CI* = confidence interval, *Ref* = reference group. Prevalence ratios indicate changes in the prevalence of the frailty in each SMI, compared to the no-SMI group, compared to the base outcome (those not frail).

Confounders based on further work demonstrated in Supplementary material K:

- Hambrecht, M., Maurer, K., Häfner, H. and Sartorius, N., 1992. Transnational stability of gender differences in schizophrenia? An analysis based on the WHO study on determinants of outcome of severe mental disorders. *European Archives of Psychiatry and Clinical Neuroscience*, *242*, pp.6-12.
- Gordon, E.H., Peel, N.M., Samanta, M., Theou, O., Howlett, S.E. and Hubbard, R.E., 2017. Sex differences in frailty: a systematic review and meta-analysis. *Experimental gerontology*, *89*, pp.30-40.
- Muntaner, C., Eaton, W.W., Miech, R. and O’campo, P., 2004. Socioeconomic position and major mental disorders. *Epidemiologic reviews*, *26*(1), pp.53-62.
- Majid, Z., Welch, C., Davies, J. and Jackson, T., 2020. Global frailty: the role of ethnicity, migration and socioeconomic factors. *Maturitas*, *139*, pp.33-41
- Health and Deprivation P Townsend P Phillimore A Beattie Health and Deprivation Published by Croom Helm 212pp 0-7099-4351-2 Nurs Stand. 1988 Jan 30;2(17):34. doi: 10.7748/ns.2.17.34.s66. PMID: 27415096.
- Wang, J. and Hulme, C., 2021. Frailty and socioeconomic status: a systematic review. Journal of Public Health Research, 10(3), pp.jphr-2021

**Supplementary Material I:**

**Figure:** Prevalence ratio estimates for the Frailty Index from unadjusted models and models adjusted for age, sex, ethnicity and country of birth


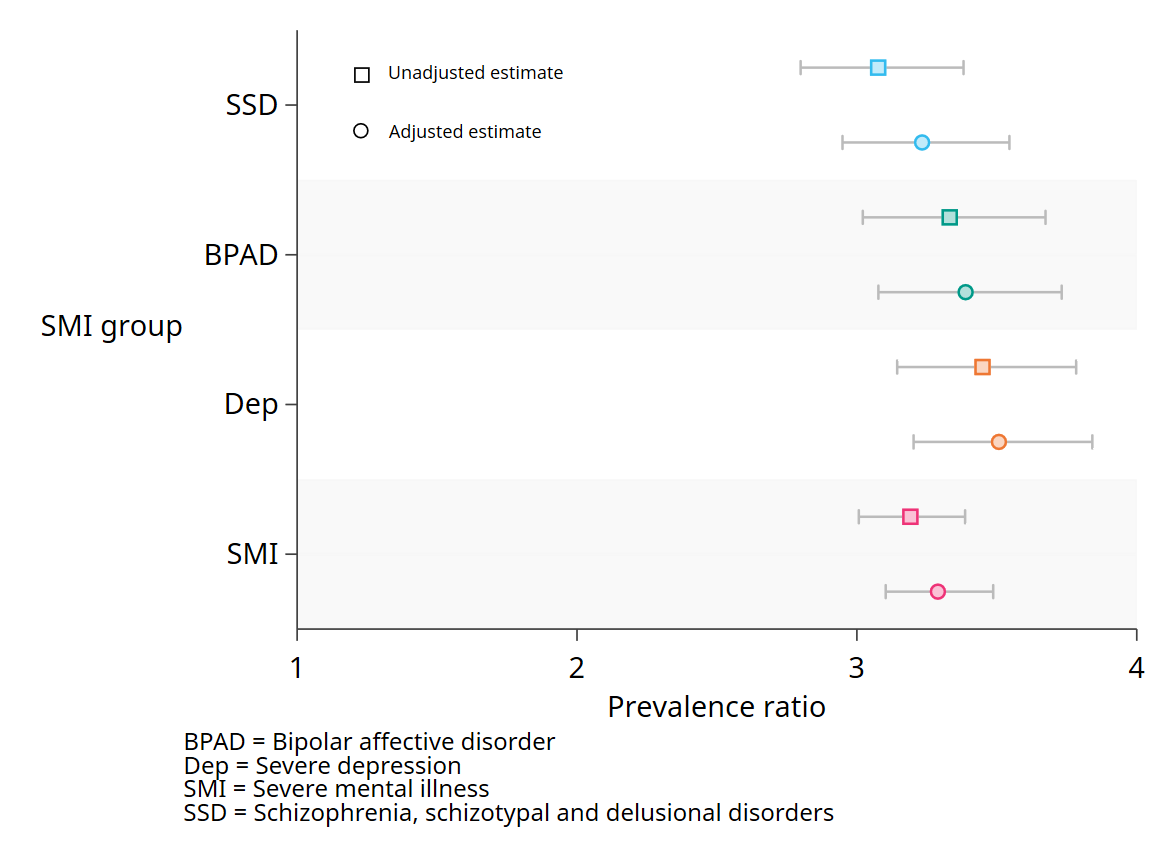


**Supplementary Material J:**

**Table:** Increased prevalence of frailty in each severe mental illness group vs no severe mental illness, from binary logistic regressions with odds ratios converted to prevalence ratios, UK Biobank baseline assessment

| Comparison | Adjusted prevalence as a fraction [95% CIs] | *SE* ^1^ | PR  [95% CIs] | *SE* ^1^ | PD  [95% CIs] | *SE* ^1^ | *P* value ^2^ | *P* value ^3^ |
| --- | --- | --- | --- | --- | --- | --- | --- | --- |
| Model 1 |  |  |  |  |  |  |  |  |
| SSD | 0.34  [0.31 to 0.38] | 0.016 | 3.23  [2.95 to 3.55] | 0.15 | 0.24  [0.21 to 0.27] | 0.016 | < 0.0005 | < 0.0005 |
| No SMI | 0.11  [0.10 to 0.11] | 0.0004 |  |  |  |  |  |  |
| Model 2 |  |  |  |  |  |  |  |  |
| BPAD | 0.36  [0.33 to 0.40] | 0.018 | 3.39  [3.08 to 3.73] | 0.17 | 0.26  [0.22 to 0.29] | 0.018 | < 0.0005 | < 0.0005 |
| No SMI | 0.11  [0.10 to 0.11] | 0.0004 |  |  |  |  |  |  |
| Model 3 |  |  |  |  |  |  |  |  |
| Dep | 0.37  [0.34 to 0.41] | 0.017 | 3.51  [3.20 to 3.84] | 0.16 | 0.27  [0.23 to 0.30] | 0.017 | < 0.0005 | < 0.0005 |
| No SMI | 0.11  [0.10 to 0.11] | 0.0004 |  |  |  |  |  |  |
| Model 4 |  |  |  |  |  |  |  |  |
| SMI | 0.35  [0.33 to 0.37] | 0.01 | 3.29  [3.10 to 3.49] | 0.10 | 0.24  [0.22 to 0.26] | 0.010 | < 0.0005 | < 0.0005 |
| No SMI | 0.11  [0.10 to 0.11] | 0.0004 |  |  |  |  |  |  |

Each model was adjusted for age (2 fractional polynomial terms, to the powers of -2 and 3), sex, country of birth and ethnicity.

^1^ Standard errors were calculated using the delta-method. ^2^ This *P* value was from a linear test of equivalence between the prevalence of the no SMI and the SMI groups. ^3^ This *P* value was from a nonlinear test that the natural log of the ARR equals 0. The 95% confidence interval for the ARR was estimated first on the log scale before exponentiating the endpoints.

**Supplementary Material K:**

**Assumption testing**

No combinations of categorical variables had 20% or more of cells with expected cell frequencies less than 5. GAMs indicated that age, the only continuous variable in the model, did not have logit linearity in any of the three models for each SMI. We therefore included fractional polynomial (fp) terms for age based on the most optimal model from 44 fp models. Two terms to the powers of 3 and 3 were selected during the multinomial logistic regression estimation process. In three separate runs, all variance inflation factors (VIF) were acceptable, except for the two age terms. All models had a mean VIF of 3.32, also not of concern. We detected no multivariate outliers in any of the three models using the BACON algorithm. As the prevalence of differing levels of frailty was greater than 10% in all analyses, we converted the estimated prevalence odds ratios to prevalence ratios for presentation.

## Analysis assumption 1 – Minimum expected cell frequencies

No more than 20% of expected cell frequencies under 5 for all pairs of categorical variables, including the dependent variable.

### Frailty Index

#### Stata code

tab2 fi4catv2 sex country_of_birth ethnicity /*

*/ household_income smi_all if fi4catv2!=.|sex!=.|country_of_birth!=.|ethnicity!=.| /*

*/ household_income!=.|smi_all!=., nofr expected

Table: Percentage of expected cell frequencies less than 5 for the ordinal logistic regression with the Frailty Index

|  | **Frailty Index** | **Sex** | **Country of birth** | **Ethnicity** | **Household income** | **Severe mental illness indicator variable** |
| --- | --- | --- | --- | --- | --- | --- |
| **Sex** | 0% |  | 0% | 0% | 0% | 0% |
| **Country of birth** | 0% | 0% |  | 0% | 0% | 0% |
| **Ethnicity** | 0% | 0% | 0% |  | 0% | 0% |
| **Household income** | 0% | 0% | 0% | 0% |  | 0% |
| **Severe mental illness indicator variable** | 0% | 0% | 0% | 0% | 0% |  |

### Physical Frailty Phenotype

#### Stata code

tab2 pfp sex country_of_birth ethnicity /*

*/ household_income smi_all if pfp!=.|sex!=.|country_of_birth!=.|ethnicity!=.| /*

*/ household_income!=.|smi_all!=., nofr expected

Table: Percentage of expected cell frequencies less than 5 for the ordinal logistic regression with the Physical Frailty Phenotype

|  | Physical Frailty Phenotype | Sex | Country of birth | Ethnicity | Household income | Severe mental illness indicator variable |
| --- | --- | --- | --- | --- | --- | --- |
| Physical Frailty Phenotype |  | 0% | 0% | 0% | 0% | 0% |
| Sex | 0% |  | 0% | 0% | 0% | 0% |
| Country of birth | 0% | 0% |  | 0% | 0% | 0% |
| Ethnicity | 0% | 0% | 0% |  | 0% | 0% |
| Household income | 0% | 0% | 0% | 0% |  | 0% |
| Severe mental illness indicator variable | 0% | 0% | 0% | 0% | 0% |  |

### Hospital Frailty Risk Score

#### Stata code

tab2 hfrs_ord_cca sex country_of_birth ethnicity /*

*/ household_income smi_all if hfrs_ord_cca!=.|sex!=.|country_of_birth!=.|ethnicity!=.| /*

*/ household_income!=.|smi_all!=., nofr expected

Table: Percentage of expected cell frequencies less than 5 for the ordinal logistic regression with the Hospital Frailty Risk Score

|  | Hospital Frailty Risk Score | Sex | Country of birth | Ethnicity | Household income | Severe mental illness indicator variable |
| --- | --- | --- | --- | --- | --- | --- |
| Hospital Frailty Risk Score |  | 0% | 0% | 6.7% - One expected frequency was 3.5 | 0% | 0% |
| Sex | 0% |  | 0% | 0% | 0% | 0% |
| Country of birth | 0% | 0% |  | 0% | 0% | 0% |
| Ethnicity | 0% | 0% | 0% |  | 0% | 0% |
| Household income | 0% | 0% | 0% | 0% |  | 0% |
| Severe mental illness indicator variable | 0% | 0% | 0% | 0% | 0% |  |

## Analysis assumption 2 – No multicollinearity

### Frailty Index

#### Stata syntax

. ssc install collin

collin calculated_age sex country_of_birth ethnicity /*

*/ household_income smi_all if fi4catv2!=. & sex!=. & /*

*/ country_of_birth!=. & ethnicity!=. & /*

*/ household_income!=. & smi_all!=.

Table: Collinearity diagnostics for the ordinal logistic regression with the Frailty Index

(obs= 353,692)

| Variable | VIF | SQRT VIF | Tolerance | R-squared | Eigenvalue | Condition Index |
| --- | --- | --- | --- | --- | --- | --- |
| Calculated age | 1.2 | 1.1 | 0.9 | 0.1 | 4.9 | 1.0 |
| Sex | 1.0 | 1.0 | 1.0 | 0.0 | 1.0 | 2.2 |
| Country of birth | 1.2 | 1.1 | 0.9 | 0.1 | 0.5 | 3.1 |
| Ethnicity | 1.2 | 1.1 | 0.9 | 0.1 | 0.3 | 3.9 |
| Household income | 1.2 | 1.1 | 0.9 | 0.1 | 0.2 | 5.4 |
| Severe mental illness | 1.0 | 1 | 1.0 | 0.0 | 0.1 | 6.6 |
| Mean VIF | 1.1 |  |  |  | 0.0 | 26.0 |
|  |  |  |  |  | Condition number ^a^ | 26.0 |
| Eigenvalues & Condition Index computed from scaled raw sscp (w/ intercept)  Detail(correlation matrix) | | | | | | 0.7 |

Notes. ^a^ Removing age reduced the condition number to 7.9, an acceptable number (threshold under 10),

Yet we retained age in the models as VIF and tolerance were acceptable.

### Physical Frailty Phenotype

#### Stata syntax

collin calculated_age sex country_of_birth ethnicity /*

*/ household_income smi_all if pfp!=. & calculated_age!=. /*

*/ & sex!=. & country_of_birth!=. & ethnicity!=. & /*

*/ household_income!=. & smi_all!=.

Table: Collinearity diagnostics for the ordinal logistic regression with the Physical Frailty Phenotype

(obs=418,612)

| Variable | VIF | SQRT VIF | Tolerance | R-squared | Eigenvalue | Condition Index |
| --- | --- | --- | --- | --- | --- | --- |
| Calculated age | 1.1 | 1.1 | 0.9 | 0.1 | 4.9 | 1.0 |
| Sex | 1.0 | 1.0 | 1.0 | 0.0 | 1.0 | 2.2 |
| Country of birth | 1.2 | 1.1 | 0.8 | 0.2 | 0.5 | 3.0 |
| Ethnicity | 1.2 | 1.1 | 0.8 | 0.2 | 0.3 | 3.8 |
| Household income | 1.1 | 1.1 | 0.9 | 0.1 | 0.2 | 5.3 |
| Severe mental illness | 1.0 | 1.0 | 1.0 | 0.0 | 0.1 | 6.4 |
| Mean VIF | 1.1 |  |  |  | 0.0 | 25.7 |
|  |  |  |  |  | Condition number ^a^ | 25.7 |
| Eigenvalues & Condition Index computed from scaled raw sscp (w/ intercept)  Detail(correlation matrix) | | | | | | 0.7 |

Notes. ^a^ Removing age reduced the condition number to 7.7, an acceptable number (threshold under 10),

Yet we retained age in the models as VIF and tolerance were acceptable.

### Hospital Frailty Risk Score

#### Stata syntax

collin calculated_age sex country_of_birth ethnicity /*

*/ household_income smi_all if calculated_age!=. & /*

*/ sex!=. & country_of_birth!=. & ethnicity!=. & /*

*/ household_income!=. & smi_all!=.

Table: Collinearity diagnostics for the ordinal logistic regression with the Hospital Frailty Risk Score

(obs=423,905)

| Variable | VIF | SQRT VIF | Tolerance | R-squared | Eigenvalue | Condition Index |
| --- | --- | --- | --- | --- | --- | --- |
| Calculated age | 1.1 | 1.1 | 0.9 | 0.1 | 4.8 | 1.0 |
| Sex | 1.0 | 1.0 | 1.0 | 0.0 | 1.0 | 2.2 |
| Country of birth | 1.2 | 1.1 | 0.8 | 0.2 | 0.5 | 3.0 |
| Ethnicity | 1.2 | 1.1 | 0.8 | 0.2 | 0.3 | 3.8 |
| Household income | 1.1 | 1.1 | 0.9 | 0.1 | 0.2 | 5.3 |
| Severe mental illness | 1.0 | 1.0 | 1.0 | 0.0 | 0.1 | 6.4 |
| Mean VIF | 1.1 |  |  |  | 0.0 | 25.6 |
|  |  |  |  |  | Condition number ^a^ | 25.6 |
| Eigenvalues & Condition Index computed from scaled raw sscp (w/ intercept)  Detail(correlation matrix) | | | | | | 0.7 |

Notes. ^a^ Removing age reduced the condition number to 7.7, an acceptable number (threshold under 10),

Yet we retained age in the models as VIF and tolerance were acceptable.

## Analysis assumption 3 – No outliers

### Stata code and output

. ssc install bacon

#### Frailty Index

#### . bacon fi4catv2 calculated_age sex country_of_birth ethnicity /*

#### > */ household_income smi_all, generate(fi_outliers) percentile(15)

#### Total number of observations: 353692

#### BACON outliers (p = 0.15): 882

#### Non-outliers remaining: 352810

#### Physical Frailty Phenotype

#### . bacon pfp sex calculated_age country_of_birth ethnicity /*

#### > */ household_income smi_all, generate(pfp_outliers) percentile(15)

#### Total number of observations: 418612

#### BACON outliers (p = 0.15): 869

#### Non-outliers remaining: 417743

#### Hospital Frailty Risk Score

. bacon hfrs_ord_cca sex country_of_birth ethnicity /*

> */ household_income smi_all, /*

> */ generate(hfrs_outliers) percentile(15)

Total number of observations: 423905

BACON outliers (p = 0.15): 0

Non-outliers remaining: 423905

## Analysis assumption 4 – Proportional odds

. ssc install omodel

. foreach v of varlist fi4catv2 pfp hfrs_ord_cca{

2. omodel logit `v' calculated_age sex country_of_birth ///

> ethnicity household_income smi_all

3. }

### Frailty Index

Approximate likelihood-ratio test of proportionality of odds

across response categories:

chi2(12) = 2501.97

Prob > chi2 = 0.0000

### Physical Frailty Phenotype

Approximate likelihood-ratio test of proportionality of odds

across response categories:

chi2(6) = 2942.79

Prob > chi2 = 0.0000

### Hospital Frailty Risk Score

Approximate likelihood-ratio test of proportionality of odds

across response categories:

chi2(6) = 115.48

Prob > chi2 = 0.0000

### Constraining odds to be proportional

We used the gologit2 Stata module to run the ordered logistic regression without assuming proportional odds. We first ran the autofit option to determine which odds required constraining over levels of the dependent variable. The autofit option indicated that ethnicity and the severe mental illness indicator variable were not proportional across different levels of the Frailty Index. We therefore constrained both variables. We needed to constrain the odds of ethnicity, country of birth and age to be proportional in the Physical Frailty Phenotype analysis, and we constrained the odds of age in the Hospital Frailty Risk Score analysis.

## Generalised ordered logistic regression estimates

### Frailty Index

Table: Generalised ordered logistic regression for the Frailty Index

|  | **Odds ratio** | ***SE*** | **Z** | ***P* value** | **95% CI LL** | **95% CI UL** |
| --- | --- | --- | --- | --- | --- | --- |
| **Relatively fit (≤0.03) vs**  **Less fit (0.03 < Frailty Index ≤ 0.10),**  **least fit (0.10 < Frailty Index ≤ 0.21) and frail (> 0.21)** |  |  |  |  |  |  |
| Age | 1.02 | 0.001 | 30.9 | < 0.005 | 1.02 | 1.02 |
| Sex | 0.83 | 0.009 | -17.1 | < 0.005 | 0.81 | 0.85 |
| Country of birth | 0.98 | 0.004 | -5.1 | < 0.005 | 0.97 | 0.99 |
| Ethnicity | 1.04 | 0.006 | 6.2 | < 0.005 | 1.03 | 1.05 |
| Household income | 0.78 | 0.004 | -49.6 | < 0.005 | 0.77 | 0.79 |
| SMI status - yes | 2.97 | 0.153 | 21.2 | < 0.005 | 2.69 | 3.29 |
| Constant | 5.3 | 0.252 | 34.7 | < 0.005 | 4.8 | 5.8 |
| **Relatively fit (≤0.03) and less fit (0.03 < Frailty Index ≤ 0.10) vs**  **least fit (0.10 < Frailty Index ≤ 0.21) and frail (> 0.21)** |  |  |  |  |  |  |
| Age | 1.01 | 0.000 | 30.5 | < 0.005 | 1.01 | 1.01 |
| Sex | 0.89 | 0.006 | -17.1 | < 0.005 | 0.88 | 0.90 |
| Country of birth | 1.00 | 0.003 | -1.9 | 0.06 | 0.99 | 1.00 |
| Ethnicity | 1.04 | 0.006 | 6.2 | < 0.005 | 1.03 | 1.05 |
| Household income | 0.73 | 0.002 | -99.1 | < 0.005 | 0.73 | 0.74 |
| SMI status - yes | 2.97 | 0.153 | 21.2 | < 0.005 | 2.69 | 3.29 |
| Constant | 0.98 | 0.030 | -0.6 | 0.5 | 0.92 | 1.04 |
| **Relatively fit (≤0.03),**  **Less fit (0.03 < Frailty Index ≤ 0.10) and least fit (0.10 < Frailty Index ≤ 0.21)**  **vs frail (> 0.21)** |  |  |  |  |  |  |
| Age | 1.01 | 0.001 | 10.6 | < 0.005 | 1.01 | 1.01 |
| Sex | 0.90 | 0.011 | -9.2 | < 0.005 | 0.88 | 0.92 |
| Country of birth | 1.00 | 0.004 | 0.1 | 0.9 | 0.99 | 1.01 |
| Ethnicity | 1.04 | 0.006 | 6.2 | < 0.005 | 1.03 | 1.05 |
